# Mediating effect of reflection types: feedback on reflection-for-action and student perception of patient-centredness

**DOI:** 10.1080/10872981.2022.2127166

**Published:** 2022-10-02

**Authors:** Hyoseon Choi, Seonyoung Jang

**Affiliations:** a Department of Medical Education, Chosun University College of Medicine, Gwangju, Republic of Korea; b Department of Liberal Art and Science, Mokpo National Maritime University, Mokpo, Republic of Korea

**Keywords:** Reflection, feedback, patient-centredness, mediation effect, reflection-for-action

## Abstract

We aimed to examine the mediating effects of reflection on the relationship between feedback for reflection-for-action (RfA-feedback) and students’ perception of the importance of patient-centred communication (PCC) education. A survey was conducted with 358 medical students and the mediation effects were analysed by performing multiple regression analysis and Sobel test. Three types of reflection (i.e., reflection-in-action, reflection-on-action, and reflection-for-action) partially mediate the relationship between feedback for reflection-for-action, and the perceived importance of PCC education. Based on these findings, the study suggests the importance of providing feedback on reflection-for-action. Such feedback can encourage student reflection, and is crucial for their future, as medical professionals.

## Introduction

Patient-centred medical education enables medical students to respond sensitively to current and future patients’ needs [1]. Recently, medical schools have begun to increase their emphasis on the importance of patient-centredness, and enhance the effectiveness of patient-centred medical education [1]. There are two important aspects for patient-centred medical education. First, students can simultaneously improve their professionalism, and their ability to provide patient-centred care [2]. Second, a patient-centred medical education encourages students to develop their self-image, on the job, as a professional [3].

The Academy of Medical Educators also supported the importance of patient-centred medical education, in their report of the five values this kind of education provides. These include medical knowledge, systems-based practice, interpersonal and communication skills, practice-based learning and improvement, and medical professionalism [2]. All these values have an impact on professionalism and increases the significance of practicing patient-centred care education.

A crucial area of patient-centred medical education is patient-centred communication (PCC). A doctor diagnoses and prescribes a patient’s symptoms, through a one-to-one conversation with the patient. Therefore, doctors need to communicate appropriately with patients. Developing a relationship through effective communication is the first step a doctor takes to treat a patient’s disease [4].

In the context of the significance of PCC, this study will attempt to analyse ways for medical students to focus on patient-centred education. One way to increase the effectiveness of patient-centred medical education is to identify the students’ perceived importance of such education. Students’ perceived importance of patient-centred communication (PCC) facilitates their motivation for a patient-centred attitude [5,6]. Meanwhile, reflection plays a vital role in a medical student’s ability to build patient-centred attitudes and relationships with patients [7]. Reflection is the deliberate consideration of one’s experiences [8]. Boud, Keogh, and Walker [9] defined it as ‘an intellectual and affective activity in which individuals explore their experiences to understand and appreciate new knowledge (p.19)’. Medical students can recognize their shortcomings by applying their acquired knowledge and skills, to analyse their interactions with patients. At this point, nurturing doctors with a patient-centred attitude, by promoting PCC through reflection, is essential in medical education [7].

Schon [10] emphasized that students should engage in reflecting on their acquired knowledge, during problem solving tasks, to ultimately achieve their desired goals. Reflection is mainly divided into three types – reflection-in-action, reflection-on-action, and reflection-for-action [10]. ‘Reflection in action’ takes place during a learning process and involves previously acquired knowledge. It usually occurs while learning or performing a task. For example, when jazz musicians improvise, they reflect on the music they create with other members, while performing. ‘Reflection on action’ occurs after an act of learning or performance. This is retrospective in nature, and refers to the thinking process used to improve something, after an action has taken place. For example, in the case of the musicians, this type of reflection includes looking back on their performance, and identifying areas to strengthen and improve. According to the conclusion of a study conducted by Perini, Cattaneo, and Tacconi [11], it was shown that the group who made the video-interview, by inserting video annotations, had a deeper reflection than the group that made the video-interview. The reflections by the group who annotated the video corresponds to ‘reflection on action’. Last, ‘reflection-for-action’ occurs when students think about what to improve for their future learning. For example, if a person ponders on their actions and process of learning in the future, long after graduation, they are engaging in reflection-for-action.

How can students be encouraged to reflect on their learning, and perceive the importance of patient-centred education? One important way to promote student reflection is to have instructors provide feedback [8]. Feedback is defined as information that promotes learning [12] regarding aspects of student’s actions or performance [13]. Instructor’s feedback help students in improving their thinking skills and self-regulated learning [14–16]. Without feedback, students may make erroneous decisions, that will ultimately lead to a poor performance [17,18]. Lala et al. [4] stated that feedback from others has a positive effect on improving medical students’ communication skills. In other words, students perform better when instructors provide feedback [19]. One implication, based on the existing literature on feedback and reflection, is that the purpose for providing feedback is essential. Ellis and Davidi [20] emphasized that students should actively reflect on their performance in order to learn from their past performance. According to Anseel, Lievens, and Schollaert [19], when instructors provide specific reasons for the successes or failures of a student’s performance, they might be able think of a variety of different strategies, and/or might review their wrong strategies for better performance.

In short, instructors should provide feedback to medical students on their communication with patients, to promote student reflection, and enable a better performance. However, only few studies have explored the relationship between instructor feedback and student reflection.

This study proposes to improve patient-centred medical education, by examining the mediating effect of the reflection types on the relationship between the purpose of feedback for reflection-for-action and students’ perceived importance of PCC education. The research questions are as follows:

Does reflection-in-action (type 1) mediate the relationship between feedback for reflection-for-action and students’ perceived importance of PCC education?

Does reflection-on-action (type 2) mediate the relationship between feedback for reflection-for-action and students’ perceived importance of PCC education?

Does reflection-for-action (type 3) mediate the relationship between feedback for reflection-for-action and students’ perceived importance of PCC education?

## Methods

We aimed to examine the relationship between the purpose of feedback for reflection for action (RfA-feedback), type of reflection, and student’s perceived importance of PCC education, to determine if the type of reflection is a mediating variable. To achieve this, we conducted a survey with medical students. The study design was approved by the Research Ethics Committee, of the higher education institution of one of the authors (IRB No. 2–1,041,055-AB-N-01-2021-53)

### Participants

To address our research questions, we conducted an online survey with students from Medical School A, located in a metropolitan city in South Korea. Emails were sent to all students, requesting their voluntary participation in the survey. 358 (51.1%) students, out of approximately 700, responded to the questionnaire. Coffee coupons worth $10 were provided to students who completed the survey. The budget was supported by the Research Foundation of South Korea. The consent form informed students that there would be no disadvantages, and that they had the right to voluntarily participate in the study. Gender, age, and school year distributions are shown in Table 1. Most respondents were in their twenties, and a relatively even distribution was found for gender and school year.

**Table 1.** Characteristics of participants.

Variable	Sub-variable	Number of Cases(N)	Percentage (%)
Gender	Male	208	58.1
	Female	150	41.9
	Total	358	100
Year	Pre-medical Program 2^nd^ Year	54	15.1
	Medical Program 1^st^ Year	76	21.2
	Medical Program 2^nd^ Year	81	22.6
	Medical Program 3^rd^ Year	85	23.7
	Medical Program 4^th^ Year	62	17.3
	Total	358	100.0
Age Group	20s	302	84.4
	30s	56	15.6
	Total	358	100.0

A first-year student at the medical school studies basic science, and second years focus on clinical science. Third and fourth years go through a clinical clerkship. Students in the first and second years learn about patient-centred care, and communication in the classroom, through the clinical expression curriculum. Those in the third and fourth years learn patient-centred care and communication in clinical practice, with real patients.

### Instruments

Questionnaire items on the purpose of RfA-feedback, as an independent variable, included questions about how much feedback was received to induce the three types of reflection. Valuables for the instrument are shown in Table 2. This study translated and modified an instrument developed by Priddis and Rogers (2018) for these items. Additionally, this study translated and modified the instrument developed by Kember et al. [21] for items regarding the mediating variable, i.e., the type of reflection. As a result, we developed six items on the purpose of feedback, with a reliability coefficient of *Cronbach’s a*= .928. In addition, 12 items on reflection types were created, showing a reliability coefficient of *Cronbach’s a* = .933. The modified instrument was validated by two educational experts.

**Table 2.** Valuables for the instrument.

Valuables	Sub-valuables	References
Reflection Types	Reflection in action (RiA)	Priddis and Rogers (2018)
	Reflection-on-action (RoA)	
	Reflection-for-action (RfA)	[21] Priddis and Rogers (2018)
Patient centred communication (PCC)	Exchanging Information	[22]
	Fostering Healing Relationships	
	Making Decisions	
	Responding to Emotions	
	Enabling Patient Self-Management	
	Managing Uncertainty	

For questionnaire items on the mediating variable – the perceived importance of PCC education – the authors translated and modified the psychometric evaluation of PCC [22] instrument. We modified the items from this instrument, to ask how students perceived the importance of PCC education, and not about PCC itself. These items were validated, after being reviewed by an expert, with a doctorate in the field of PCC. The developed items consisted of five items for each sub-section, for a total of 30 items, with a reliability coefficient *Cronbach’s a* = .976.

A 5-point Likert scale was used, replicating the original instruments. Two medical students reviewed the developed instrument, to check if any items were difficult to understand, and ensured the use of appropriate expressions. The instrument was then revised to reflect their opinions.

### Data analysis

We performed a basic analysis of the responses collected by conducting a reliability analysis, frequency analysis, descriptive statistical analysis, and correlation analysis. In addition, we conducted a multiple regression analysis and a Sobel test, to determine relationships and analyse the mediating effect, to achieve our objectives. Specifically, we used the multiple regression analysis method proposed by Baron and Kenny [23], to conduct a mediation analysis, using SPSS Statistics 24 software.

## Results

Our study aimed to determine if feedback and reflection affect the perceived importance of PCC in medical education. In addition, we sought to determine if there is a difference in reflection type, or the perceived importance of PCC education, according to the purpose of feedback for RfA. We also aimed to determine if the reflection type mediates the relationship between the purpose of feedback for RfA, and the perceived importance of PCC education.

### Descriptive and correlation analysis among the purpose of feedback for RfA, reflection type, and student’s perceived importance of PCC education

We performed a descriptive statistical analysis of the purpose of RfA-feedback, type of reflection, and students’ perceived importance of PCC education. Results are shown in Table 3.

**Table 3.** Descriptive analysis of factors.

Variable	Sub-variable	Number of Cases (N)	Mean (M)	Standard Deviation (SD)
Feedback on reflection-for-action (RfA-feedback)		358	4.09	0.81
	Reflection-in-Action (Type 1)	358	4.17	0.67
Reflection types	Reflection-on-Action (Type 2)	358	4.16	0.67
	Reflection-for-Action (Type 3)	358	4.27	0.68
	Total	358	4.19	0.58
Perceived Importance of PCC Education		358	4.43	0.51

The descriptive statistical analysis results demonstrated that the mean distribution of responses for all variables was between 4.09 and 4.43 (standard deviation 0.51∼0.81). With reference to the purpose of the RfA-feedback, students perceived that they were provided with feedback that required them to engage in the three types of reflection. Additionally, they responded that a certain degree of reflection took place regarding all three types of reflection. Furthermore, the students tended to recognize the importance of PCC education.

We performed a correlation analysis, to determine if the three variables correlated with one another. All three were found to have a statistically significant positive correlation with each other. These results are shown in Table 4.

**Table 4.** Correlations of factors.

Correlation Coefficient		Purpose of RfA-feedback	Reflection types			Perceived Importance of PCC Education
			Reflection -in-Action	Reflection -on-Action	Reflection -for- Action	
Feedback on reflection-for-action (RfA-feedback)		1				
Reflection types	Reflection-in-Action (Type 1)	.504^a***^	1			
	Reflection-on-Action (Type 2)	.504^a***^	.606^a***^	1		
	Reflection-for-Action (Type 3)	.498^a***^	.539^a***^	.689^a***^	1	
Perceived Importance of PCC Education		.441^a***^	.518^a***^	.544^a***^	.511^a***^	1

^a^*p*< 0.05, ** *p* < 0.01, *** *p* < 0.001

### The mediating effect of reflection-in-action (Type 1) between the purposes of RfA-feedback and perceived importance of PCC education

We aimed to examine the mediating effect of the reflection-in-action, on the relationship between the purpose of RfA-feedback, and the perceived importance of PCC education. We reviewed this mediating effect, according to Baron and Kenny’s [23] three-step process of testing mediation. Our results are as shown in Table 5.

**Table 5.** The mediating effect of reflection-in-action.

Step	Dependent Variable	Explanatory Variable	B	S.E.	β	t
1	Type of Reflection 1:Reflection-in-Action	(Constant)	2.522	.152		16.539***
		Purpose of RfA-feedback	.402	.037	.504	11.002***
		*F*= 121.040*** *R^2^ *= .254 *adjR^2^ *= .252 *D-W*= 2.078				
2	Perceived Importance of PCC Education	(Constant)	3.305	0.124		26.588***
		Purpose of RfA-feedback	0.276	0.030	0.441	9.260***
		*F*= 85.743*** *R^2^ *= .194 *adjR^2^ *= .192 *D-W*= 2.012				
3	Perceived Importance of PCC Education	(Constant)	2.521	.153		16.476***
		Purpose of RfA-feedback	.151	.032	.241	4.727***
		Type of Reflection 1:Reflection-in-Action	.311	.040	.396	7.769***
		*F*= 80.194*** *R^2^ *= .311 *adjR^2^ *= .307 *D-W*= 1.993				

First, for the independent variables, the purpose of feedback regarding reflection-for-action had a statistically significant effect on the reflection-in-action, which were the mediating variables. Second, we discovered that the purpose of RfA-feedback – the independent variable – affects the perceived importance of PCC education – the dependent variable. That is to say, the purpose of the RfA-feedback was found to affect the perceived importance of PCC education. Third, reflection-in-action, a mediating variable, was found to affect the perceived importance of PCC education, the dependent variable. Moreover, the β, which is the standardization coefficient of the effect of the independent variable, on the dependent variable in Step 3, was smaller than the β in Step 2. This indicates the partial mediating effect of reflection-in-action.

**Figure 1.**
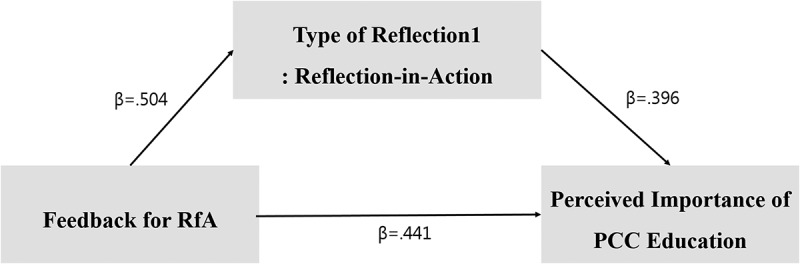
Partial mediating effect model of reflection-in-action (Type 1).

Thus, we found that reflection-in-action partially mediates the relationship between the purpose of RfA-feedback, and the perceived importance of PCC education. Moreover, the *Sobel test* was performed to confirm the mediation effect. The Sobel test statistic (z) was found to be significant (z = 6.323, p < .000). Figure 1 shows a model of the partially mediating effects of reflection-in-action (Type 1), on the effect of the RfA-feedback, on the perceived importance of PCC education.

### The mediating effect of reflection-on-action (Type 2) between the purposes of feedback for RfA and perceptions of PCC education

We also aimed to examine the mediating effect of the reflection-on-action, on the relationship between the purpose of RfA-feedback and the perceived importance of PCC education. We reviewed this mediating effect, and the results are as shown in Table 6.

**Table 6.** The mediating effect of reflection-on-action.

Step	Dependent Variable	Explanatory Variable	B	S.E.	β	t
1	Type of Reflection 2:Reflection-on-Action	(Constant)	2.456	.158		15.592***
		Purpose of RfA-feedback	.416	.038	.504	11.012***
		*F*= 121.254*** *R^2^ *= .254 *adjR^2^ *= .252 *D-W*= 2.054				
2	Perceived Importance of PCC Education	(Constant)	3.305	0.124		26.588***
		Purpose of RfA-feedback	0.276	0.030	0.441	9.260***
		*F*= 85.743*** *R^2^ *= .194 *adjR^2^ *= .192 *D-W*= 2.012				
3	Perceived Importance of PCC Education	(Constant)	2.499	.147		17.019***
		Purpose of RfA-feedback	.140	.031	.223	4.442***
		Type of Reflection 2:Reflection-on-Action	.328	.038	.432	8.606***
		*F*= 88.706*** *R^2^ *= .333 *adjR^2^ *= .329 *D-W*= 1.960				

A three-step multiple regression analysis shows the purpose of the RfA-feedback as the independent variable, and reflection-on-action as the mediating variable, affecting students’ perceived importance of PCC education, as the dependent variable. Moreover, the β, which is the standardization coefficient, of the effect of the independent variable on the dependent variable in Step 3, was smaller than the β in Step 2, indicating a partially mediating effect of reflection-on-action.

**Figure 2.**
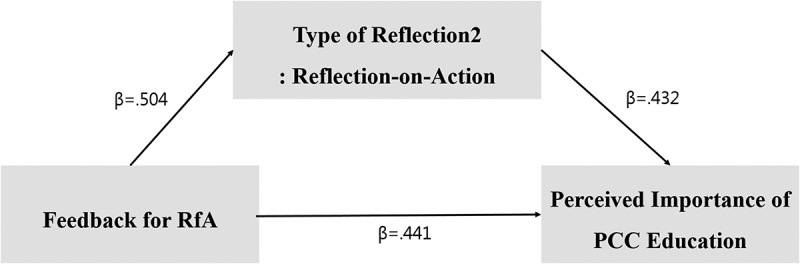
Partial mediating effect model of reflection-on-action (Type 2).

Therefore, we found that reflection-on-action partially mediates the relationship between the purpose of the RfA-feedback, and the perceived importance of PCC education. Moreover, the Sobel test statistic (z) was found to be significant (z = 6.778, p < .000). Figure 2 shows a model of the partially mediating effects, of reflection-on-action (Type 2), on the effect of RfA-feedback on the perceived importance of PCC education.

### The mediating effect of reflection-for-action (Type3) between the purposes of feedback for RfA and perceptions of PCC education

Last, we also aimed to examine the mediating effect of the reflection-for-action, on the relationship between the purpose of RfA-feedback and the perceived importance of PCC education. We reviewed this mediating effect, and the results are as shown in Table 7.

**Table 7.** The mediating effect of reflection-for-action.

Step	Dependent Variable	Explanatory Variable	B	S.E.	β	t
1	Type of Reflection 3:Reflection-for-Action	(Constant)	2.563	.160		15.993***
		Purpose of Feedback for RfA	.417	.038	.498	10.848***
		*F*= 117.680*** *R^2^ *= .248 *adjR^2^ *= .246 *D-W*= 2.056				
2	Perceived Importance of PCC Education	(Constant)	3.305	0.124		26.588***
		Purpose of Feedback for RfA	0.276	0.030	0.441	9.260***
		*F*= 85.743*** *R^2^ *= .194 *adjR^2^ *= .192 *D-W*= 2.012				
3	Perceived Importance of PCC Education	(Constant)	2.561	.151		16.925***
		Purpose of Feedback for RfA	.155	.032	.248	4.856***
		Type of Reflection 3:Reflection-for-Action	.290	.038	.387	7.599***
		*F*= 78.580*** *R^2^ *= .307 *adjR^2^ *= .303 *D-W*= 2.083				

A three-step multiple regression analysis shows the purpose of RfA-feedback as the independent variable, and reflection-for-action as the mediating variable that affect students’ perceived importance of PCC education, the dependent variable. Moreover, the β, which is the standardization coefficient, of the effect of the independent variable on the dependent variable in Step 3, was smaller than the β in Step 2. This indicates a partial mediating effect of reflection-for-action.

**Figure 3.**
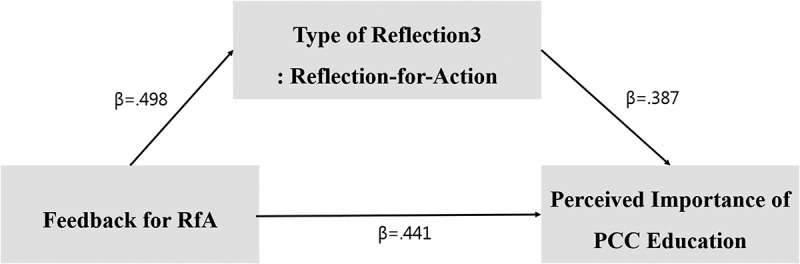
Partial mediating effect model of reflection-for-action (Type 3).

Thus, we found that reflection-for-action partially mediates the relationship between the purpose of RfA-feedback, and the perceived importance of PCC education. Moreover, the Sobel test statistic (z) was found to be significant (z = 6.265, p < .000). Figure 3 shows the model of the partially mediating effects of reflection-for-action (Type 3), on the effect of RfA-feedback on the perceived importance of PCC education.

## Discussion

Our results revealed that reflection completely mediates the relationship between the purpose of RfA-feedback, and the perceived importance of PCC. Based on these findings, we will discuss the implications regarding what feedback for RfA and reflection will strengthen students’ reflection.

### The effect of future-focused feedback on student reflection

According to the results, feedbacks that is provided for the purpose of reflection-for-action promotes student reflection. Additionally, Jonsson’s [24] study reported that students prefer helpful feedback, and they prefer information that can be used in the near future. Moreover, a number of studies [25–39] support the importance of providing constructive feedback, for the future improvement of students.

The role of feedback is to help students improve their performance and competency in the future. There is a difference in time between when feedback is given, and when its effect appears. Considering the results of previous studies and this study, it can be concluded that instructors should consider students’ future improvement when providing feedback.

In fact, Keller [40] argued that motivation plays a crucial role in students’ successful learning, and established the following six indicators of motivation: (1) the desire to succeed; (2) encouragement and need for learning; (3) future hopes and ideals; (4) appreciation of the learning process; (5) interesting and enjoyable learning activities; and (6) a conducive learning environment that allows students to learn comfortably, and well. Of these, reflection-for-action has a decisive effect on both, ‘encouragement and need for learning’ and ‘future hopes and ideals’. This is because recognizing how one’s current studies will affect one’s career in the after graduation helps students understand why they must review what they are studying, and ideal level of performance for the future.

### Reflection and medical education

We found that feedback provided for the purpose of reflection-for-action, and the types of student reflection, can positively affect students’ perceived importance of PCC education. All three types of reflection were found to affect this.

The purpose of undergraduate medical education is to train students to be become qualified doctors. Therefore, when instructors give medical students information, for the purpose of reflection-for-action, it is assumed that they will become doctors. In other words, feedback on how students should act, when they become doctors, motivates them to learn, and to think and reflect on this input in the long run.

In addition to medical schools, this could apply to different higher education institutions, when aiming to foster professionalism in their students. For example, institutions with majors that aim to cultivate professionals, such as teachers’ colleges, law schools, and art schools, could help students improve their abilities, by providing feedback for the purpose of reflection-for-action, as well as having students engage in actual reflection-for-action.

For students to recognize the necessity of patient-centred education, we found it vital for instructors to provide specific feedback that encourages reflection. This study differs from the context set by studies like, Boud [41], and Carless [27], that existing studies on feedback focus on the type and timing of the feedback. It emphasizes the instructor’s purpose in providing feedback, and determining the kind of feedback that encourages student reflection.

## Conclusion

We aimed to examine the mediating effects of reflection on the relationship between feedback for reflection-for-action (RfA-feedback) and students’ perception of the importance of patient-centred communication (PCC) education. Three types of reflection (i.e., reflection-in-action, reflection-on-action, and reflection-for-action) partially mediate the relationship between feedback for reflection-for-action, and the perceived importance of PCC education. Based on these findings, the study suggests the importance of providing feedback on reflection-for-action. Such feedback can encourage student reflection, and is crucial for their future, as medical professionals.

However, our study has a limitation, in that we derived our findings from students’ experiences, with feedback to examine their perceptions. Therefore, future studies should conduct in-depth interviews with students who received feedback, to determine its positive effect on students. Furthermore, future studies should examine strategies or guidelines for providing feedback, that will encourage student reflection, to help instructors in actively offering constructive feedback.

## Data Availability

The datasets used during the current study are available from the corresponding author on reasonable request.
